# Caspase 8 expression may determine the survival of women with ovarian cancer

**DOI:** 10.1038/cddis.2015.398

**Published:** 2016-01-14

**Authors:** M Kim, L Hernandez, C M Annunziata

**Affiliations:** 1 Women's Malignancies Branch, Center for Cancer Research, National Cancer Institute, Bethesda, MD, USA

Ovarian cancer cells frequently demonstrate resistance to apoptosis-inducing chemotherapy. The unfortunate consequence is relapse with incurable disease in over half of the women diagnosed with this disease.

Many gene signatures and protein markers have been linked to the prognosis of women with ovarian cancer. For example, the Cancer Genome Atlas and the Australian Ovarian Cancer Study identified four major subgroups of high-grade serous ovarian cancer (HGSOC) based on gene expression signatures.^1, 2^ These subgroups carried prognostic information, with the immune-related subtypes having the best prognosis, and the mesenchymal types conveying relatively shorter overall survival. Recent meta-analyses containing these datasets and gene expression profiles from eight other patient databases identified 200-gene signatures to predict either overall survival of women with HGSOC, or the ability to undergo optimal cytoreductive surgery at the time of diagnosis.^3^ These signatures provide insight into the disease process and a wide range of potential therapeutic avenues based on signaling networks described within the gene sets.

Others have focused on the ability of ovarian cancer cells to undergo apoptosis. Many cancer cells possess the ability to resist apoptosis either *de novo* at diagnosis or at the time of relapse. Mechanisms to resist apoptosis are myriad and may be related to overexpression of anti-apoptotic proteins such as those in the Bcl-2 family or down regulation of pro-apoptotic proteins. Both mechanisms have been implicated in ovarian cancer.^4, 5^

It is plausible to hypothesize, then, that evidence for cancer cell resistance to apoptosis at the time of diagnosis will result in shorter overall survival of women with these cancers. Indeed, low expression of pro-apoptotic HtrA20 or SMAC has individually been linked to poor outcome in women with ovarian cancer.^6, 7^ Negative immunohistochemical staining for HtrA2, an inhibitor of X-linked inhibitor of apoptosis (XIAP), was significantly related to shorter progression free and overall survival for women with HGSOC. Similarly, low levels of SMAC in the circulating plasma of women with ovarian cancer were also closely related to shorter overall and disease free survival. In addition, low XAF1, a negative regulator of XIAP, was also associated with higher-grade histology and worse overall survival.^8^

Findings with Caspase 3, however, are conflicting. In some cases, low cleaved Caspase 3 in the tumor cells of malignant effusions was associated with shorter overall survival.^9^ In another study, low expression of Caspase 3 gene was associated with shorter overall survival, but immunohistochemistry showed that the Caspase 3 was localized in the tissue macrophages and not in the cancer cells.^10^ In a third situation, low cleaved Caspase 3 in ovarian cancer cells identified cases with dramatically longer overall survival in women.^11^ Therefore, the prognostic value of Caspase 3 remains unclear.

Similar controversy has occurred with NF-*κ*B signaling in ovarian cancer. We previously showed that evidence of elevated NF-*κ*B signaling conveyed a poor prognosis in ovarian cancer and experimentally linked NF-*κ*B to cellular features of aggressive cancer cells such as invasion and angiogenesis.^12^ Conversely, NF-*κ*B signaling also promotes inflammation and the presence of inflammatory cells in the microenvironment of ovarian cancers is associated with better outcome.^13^ Additional work suggests that the level of NF-*κ*B activation in ovarian cancer cells may determine whether NF-*κ*B acts as a tumor suppressor or an oncogene.^14^ With this background, we sought to investigate the cancer cell specific mechanisms of NF-*κ*B signaling in ovarian cancer.

In our current paper in *CDDiscovery*, we used a genome wide RNAi to investigate factors that influence NF-*κ*B signaling in ovarian cancer.^15^ Remarkably, we found that Caspase 8 is a key regulator of pro-survival NF-*κ*B activity in this context. Looking deeper into the mechanism of this functional interaction we confirmed that Caspase 8 was critical for apoptosis triggered via the extrinsic pathway downstream of TNF*α*. Indeed, ovarian cancer patients whose tumors expressed low levels of Caspase 8 achieved shorter overall survival compared to those with higher Caspase 8 expression, in three large datasets containing gene expression profiles from women newly diagnosed with ovarian cancer. On the other hand, the loss of Caspase 8 enhanced cancer cell death via non-apopotic means, namely necroptosis. The necroptotic form of cell death was further enhanced when NF-*κ*B activity was blocked in the setting of low Caspase 8 expression.

Standard chemotherapy regimens are typically thought to rely on apoptosis for the elimination of cancer cells. Cancers with low Caspase 8, therefore, may be inherently resistant to such therapies. Ovarian cancer is a disease characterized by high rates of relapse after standard chemotherapy and therefore, in need of novel strategies to overcome chemotherapy resistance. Necroptosis-inducing therapies may provide such an avenue. A subset or a sub-population of cells with insufficient Caspase 8 may be resistant to apoptosis, but targetable via necroptosis. Caspase 8 activity could be compromised in ovarian cancer due to mutation, deletion or underexpression. The absence of Caspase 8 stabilizes RIPK1, a protein required for formation of the necrosome complex, in combination with RIPK3 and MLKL. Stabilization of this complex predisposes cells to necroptotic cell death. SMAC mimetics that target inhibitor of apoptosis (IAP) proteins for degradation, may be one approach to bypass resistance to apoptosis in Caspase 8 low tumors. The loss of both IAPs and Caspase 8, in cells where RIPK1 is stabilized, could tip the balance towards necroptosis (Figure 1).

These exciting new therapeutic approaches require prospective clinical validation. Such trials should include markers to evaluate the molecular characteristics of each woman's tumor, including sub-populations of potentially chemoresistant cells. Interference with NF-*κ*B-and IAP-driven survival effects could be advantageous in promoting necroptotic cell death in ovarian cancers that resist apoptosis. This could lead to biomarker-driven therapeutic strategies to increase the effectiveness of standard chemotherapy.

## Figures and Tables

**Figure 1:**
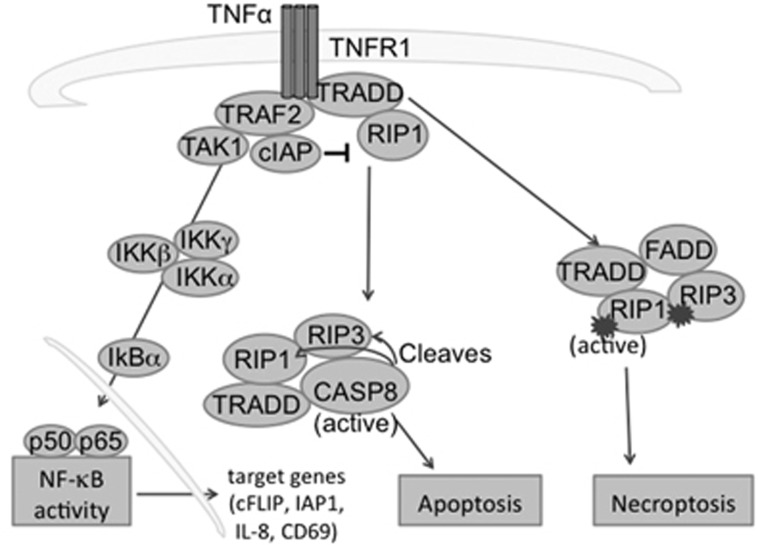
Schematic of TNF signaling. TNF*α* can trigger cell proliferation, apoptosis or necroptosis, depending on the balance of proteins within the cell. With inhibitor of apoptosis (IAP) proteins intact, NF-*κ*B can be activated and proliferation can be increased. With Caspase 8 present and NF-*κ*B blocked, cells undergo apoptosis. When both IAPs and Caspase 8 are deficient, cells undergo necroptosis
